# Supplementary material for: GNOMES: an integrated framework for genome-wide normalization and differential binding analysis of CUT&RUN and ChIP-seq data
Source: bioRxiv. 2026 Apr 21:2026.04.16.718722. Preprint. [Version 1] doi: 10.64898/2026.04.16.718722 (PMC13131678; doi:10.64898/2026.04.16.718722)

Suppl. Figure 1

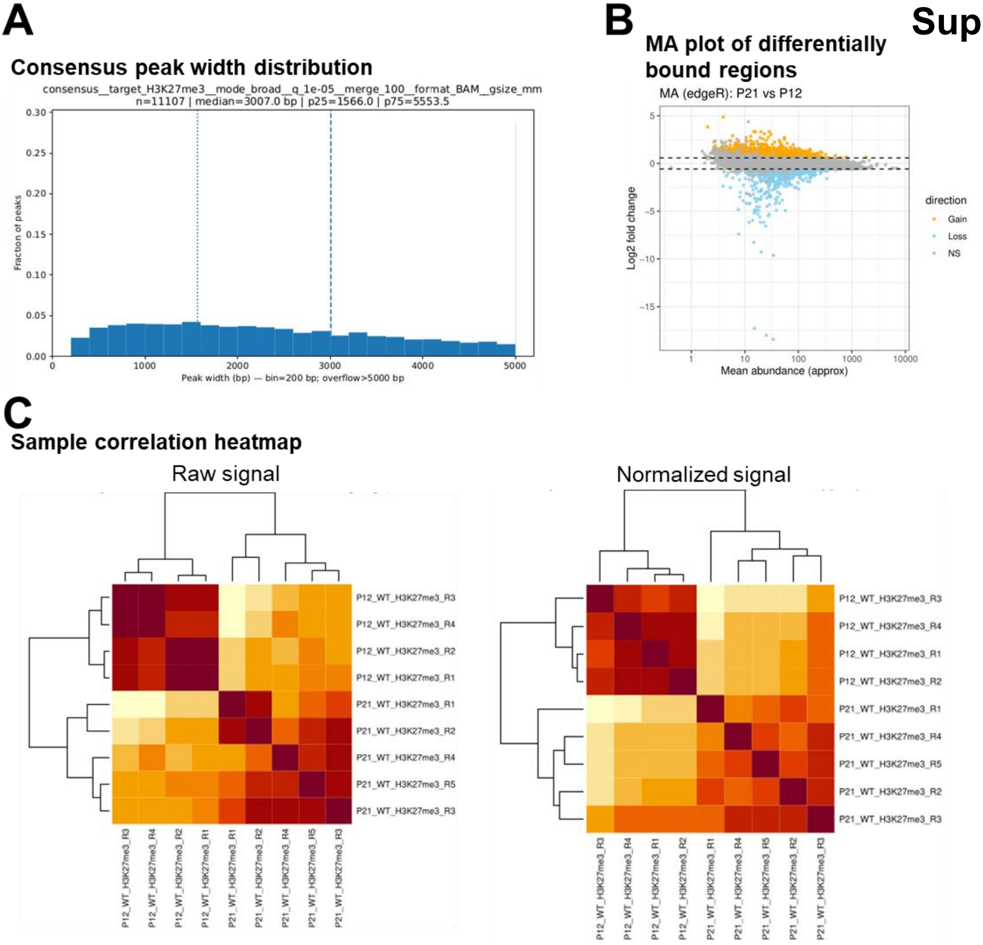

Supplement: Supplement 1 — Supplementary Figure 1. GNOMES consensus and diff modules quality control plots. (A) Peak width distribution of the consensus peak set selected (MACS2 q-value threshold of 0.00001 and a merge distance of 100bp) for downstream differential binding analysis. (B) MA plot of differentially H3K27me3 bound regions from P12 to P21. Significantly gain and loss regions are highlighted in orange and blue, respectively (edgeR, adjusted p-value < 0.05 and |log2FC| ≥ 0.58). (C) Sample correlation heatmap of signal in consensus peaks before (left) and after (right) GNOMES normalization. [file media-1.pdf]
