# Supplementary figures and images for: GNOMES: an integrated framework for genome-wide normalization and differential binding analysis of CUT&RUN and ChIP-seq data
Source: bioRxiv. 2026 Apr 21:2026.04.16.718722. Preprint. [Version 1] doi: 10.64898/2026.04.16.718722 (PMC13131678; doi:10.64898/2026.04.16.718722)

**A**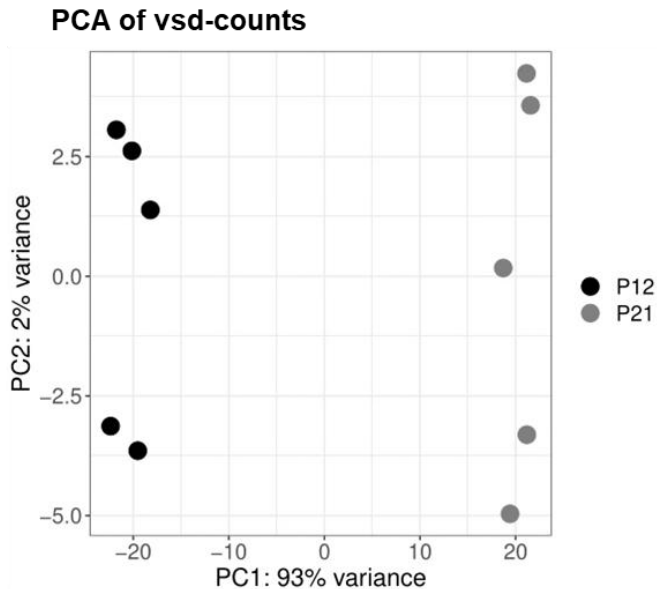**B**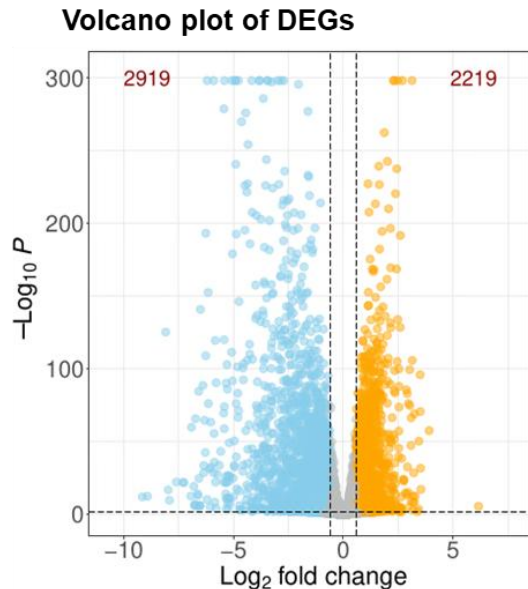

Supplement: Supplement 2 — Supplementary Figure 2. Bulk-RNAseq differential expression analysis in P12 and P21 mouse cerebellum samples. (A) Principal Component Analysis (PCA) of bulk-RNAseq samples after variance-stabilizing transformation with DESeq2. (B) Volcano plot of differentially expressed genes (DEGs) between P12 and P21. Significantly up-regulated and down-regulated genes are highlighted in orange and blue, respectively (adjusted p-value < 0.05 and |log2FC| ≥ 0.58), with the total number of DEGs indicated in red. [file media-2.pdf]
